# Journée scientifique Covid et société en Guyane et aux Antilles - 25 mars 2022 - Cayenne, Guyane

**DOI:** 10.48327/mtsi.v2i3.2022.270

**Published:** 2022-08-29

**Authors:** Marc-Alexandre TAREAU, Nicolas VIGNIER, Mayka MERGEAY-FABRE, Agnès CLERC RENAUD, Stéphanie MULOT, Guillaume ODONNE, Loïc EPELBOIN

**Affiliations:** 1 Laboratoire Écologie, évolution, interactions des systèmes amazoniens (LEEISA), 275 route de Montabo, 97300 Cayenne (UAR 3456). CNRS, Université de Guyane. IFREMER, Cayenne, Guyane, France; 2 COREVIH, Centre hospitalier de Cayenne, Avenue des flamboyants, Cayenne 97306, Guyane, France; 3 CIC INSERM 1424, Centre hospitalier de Cayenne, Avenue des flamboyants, Cayenne 97306, Guyane, France; 4 Université Toulouse-Jean-Jaurès, 5 Allée Antonio Machado, 31058 Toulouse, France; 5 Unité des Maladies infectieuses et tropicales, Centre hospitalier de Cayenne, Avenue des flamboyants, Cayenne 97306, Guyane, France

**Keywords:** Covid-19, Ethnobotanique, Anthropologie, Épidémiologie, Santé publique, Infectiologie, Hésitation vaccinale, Confinement, Guyane, Antilles françaises, Covid-19, Ethnobotany, Anthropology, Epidemiology, Public Health, Infectiology, Vaccine hesitancy, Lockdown, French Guiana, French West Indies

La journée scientifique « Covid et société en Guyane et aux Antilles », qui s'est déroulée le 25 mars 2022 à Cayenne et en distanciel, est le fruit de la collaboration entre scientifiques et chercheurs issus de divers horizons académiques, et souhaitant donner un éclairage nouveau aux conséquences majeures de l’épidémie de Covid-19 dans les territoires français d'Amérique. Ainsi, soignants et chercheurs issus de l'ethnobotanique, de l'anthropologie, de l’épidémiologie, de la santé publique et de l'infectiologie ont réuni pour une journée scientifique exceptionnelle à Cayenne des intervenants en provenance de Guyane, Martinique, Guadeloupe et de l'Hexagone, avec une approche multidisciplinaire de l’épidémie de Covid-19 dans nos territoires. La session d'ouverture a été consacrée à l’épidémiologie, avec la présentation des données par Mme Luisiane Carvalho et le Dr Cyril Rousseau, épidémiologistes de Santé publique France Antenne Guyane sur l’épidémie de Covid-19 en Guyane, suivie d'une présentation de l’état des lieux du Covid-19 au CHU de Martinique par le Pr André Cabié, chef du service de Maladies infectieuses du dit hôpital, puis la présentation de l’étude sur la transmission du Covid-19 dans la population guyanaise par M. Claude Flamand, de l’équipe d’épidémiologie de l'Institut Pasteur de la Guyane, et enfin un exposé sur la mortalité hospitalière du Covid-19 en Guadeloupe par le Dr Bruno Jarrige, le directeur médical de crise de ce territoire. La seconde session était consacrée à l'ethnomédecine, avec une présentation de M. Marc- Alexandre Tareau, post-doctorant au laboratoire LEEISA du CNRS et de l'Université de Guyane, sur l'adaptation des médecines et des phytothérapies créoles guyanaises et haïtiennes face à l’émergence du Covid-19, suivie d'une présentation d'un travail mené à l'Institut Pasteur de la Guyane par la masteurante Mme Glwadys Forsans sur l'utilisation de la pharmacopée locale et l'hésitation vaccinale en Guyane et enfin une présentation par le Dr Emmanuel Nossin, pharmacien en Martinique, au sujet des plantes médicinales de la pharmacopée antillaise pouvant être associées à la lutte contre le Covid-19. La session suivante a été consacrée aux déterminants sociaux de l'hésitation vaccinale avec en introduction une présentation du Dr Amandine Gagneux-Brunon, infectiologue au CHU de Saint-Étienne, sur l’évolution de la perception de la vaccination suivie d'une présentation du Pr Loïc Epelboin, infectiologue au Centre hospitalier de Cayenne rappelant les enjeux politiques et sociétaux de l’épidémie de Covid-19 et l'arrivée du vaccin en Guyane, suivie d'une présentation de Mme le Pr Stéphanie Mulot, sociologue à l'Université Toulouse-Jean-Jaurès sur la politisation du rapport au Covid-19 et son vaccin en Guadeloupe, puis la présentation d'une étude sur l'hésitation vaccinale des soignants de Guyane par le Dr Nicolas Vignier, chercheur en épidémiologie au Centre d'investigation clinique du CH de Cayenne et enfin l’étude sur la caractérisation et le suivi des hésitations vaccinales dans la population guyanaise par M. Claude Flamand. Enfin la session qui a clôturé cette journée était consacrée à l'impact des périodes de confinement sur les modes de vie avec une première présentation par Mme Priscilla Thébaux, doctorante en anthropologie au LEEISA, de son travail sur l'accès à l'eau potable en temps de crise sanitaire en Guyane, suivie de la présentation de Mme Margot Schneider, masteurante au LEEISA et à l'Université Paris Cité, sur les conséquences de la syndémie sur la vie des habitants de Saint-Georges-de-l'Oyapock à la frontière franco-brésilienne, pour terminer avec la présentation de Mme Frédérique Groene, psychologue et chercheuse à l'Université de Guyane sur les indices psycho-sociétaux de l’épidémie de Covid-19 en Guyane. La journée a été conclue par les invitées d'honneur, Mme le Pr Stéphanie Mulot, sociologue à l'Université Toulouse-Jean-Jaurès et Mme le Pr France Roblot-Cazenave, présidente de la Société de pathologies infectieuses de langue française (SPILF), qui ont vanté l'originalité du concept du mélange des points de vue scientifiques à la croisée de la médecine, de l’épidémiologie et des sciences sociales sur la question du Covid-19, ainsi que la qualité et la variété des présentations. Une réflexion a été initiée sur la possibilité de reproduire et pérenniser ce concept original en proposant de nouvelles journées pour les années suivantes autour d'autres thématiques transdisciplinaires: VIH, maladies du péril fécal, maladies à transmission vectorielle, qui sait?

**Figure 1:**
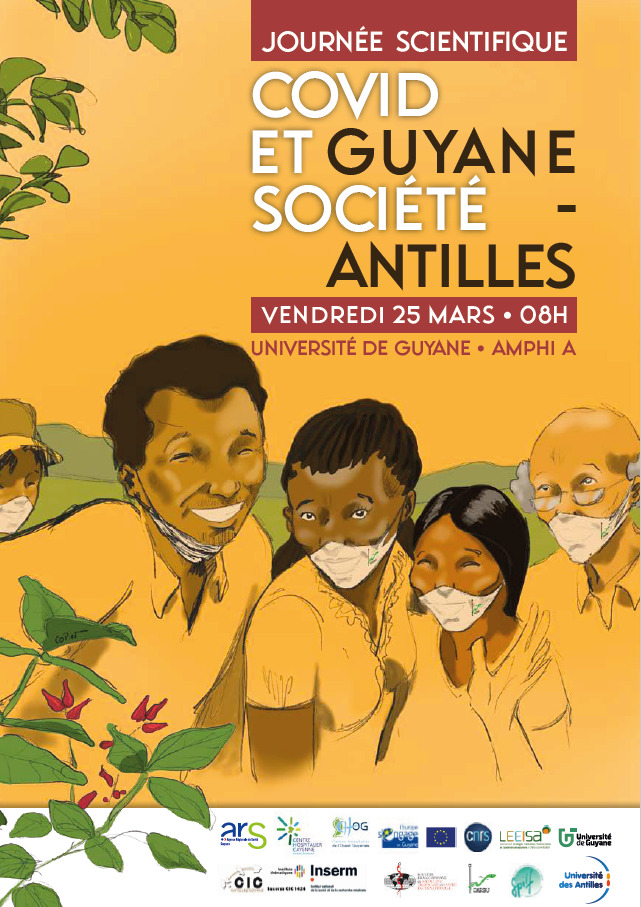
Affiche du colloque « Covid et société Guyane-Antilles » du 25 mars 2022 à Cayenne. Dessin: Olivier Copin. Infographie: Bénédicte Sauvage, Bcom Poster for the “Covid and society in French Guiana and the West Indies” conference on 25 March 2022 in Cayenne. Design: Olivier Copin. Computer graphics: Bénédicte Sauvage, Bcom

## Liens D'intérêts

Les auteurs ne déclarent aucun lien d'intérêt.

## Remerciements

Le comité d'organisation tient à remercier Mme Bénédicte Sauvage de Bcom pour toute l'organisation technique et logistique du congrès, Aéroprod pour avoir permis le suivi en distanciel, l'ARS Guyane, financeur principal de ces journées et en particulier Mme Solène Wiedner-Papin, Mme le Pr France Roblot-Cazenave, présidente de la SPILF, société savante et co-financeur, d'avoir bien voulu venir assister en présentiel à ces journées, et enfin l'Université de Guyane et son président, M. Antoine Primerose, pour avoir gracieusement mis à disposition l'amphithéâtre A du campus Troubiran. Ces journées ont été soutenues par l'Union européenne et la Collectivité territoriale de Guyane dans le cadre de l'appel à projet Flash Covid-19 et de ses programmes MELOCOVID (FEDER, SYNERGIE GY0027304) et AMAZCOV'N FEVERS (FEDER, SYNERGIE GY0028034).

