# Supplementary material for: Covid and society in French Guiana and the French West Indies Scientific day - March 25, 2022 - Cayenne, French Guiana
Source: Med Trop Sante Int. 2022 Aug 29;2(3):mtsi.v2i3.2022.270. [Article in French] doi: 10.48327/mtsi.v2i3.2022.270 (PMC9557817; doi:10.48327/mtsi.v2i3.2022.270)
Supplement: Related texts

# Research article: Épidémiologie du Covid-19 en Guyane 2020-2022

**Published:** 2022-08-29

**Authors:** Cyril ROUSSEAU, Luisiane CARVALHO

**Affiliations:** 1 Direction du Pôle des centres délocalisés de prévention et de soins, Centre hospitalier de Cayenne, Avenue des flamboyants, Cayenne 97306, Guyane, France; 2 Santé publique France en Guyane

C'est le 4 mars 2020, six semaines après les premières détections d'infections à SARSCoV-2 importées dans l'Hexagone, que la Guyane a détecté à son tour ses premiers cas importés suite au cluster dit « de Mulhouse ». Confiné comme l'Hexagone dès mi-mars, le territoire a connu une succession de clusters d'importation puis de transmissions autochtones secondaires durant 2 mois, dans un contexte de contact tracing et d'investigations approfondies. Fin avril 2020, la forte amplification de l’épidémie brésilienne a donné lieu à un passage en situation épidémique sur la frontière de l'Est guyanais, suivie d'une généralisation au territoire. Ceci témoigne de l'inscription définitive de la Guyane dans son environnement régional latinoaméricain.

Cinq vagues se sont succédé jusque début 2022, au rythme des émergences de variants, avec des impacts différents. Les dispositifs de surveillance se sont considérablement étoffés et le temps initialement gagné avant la première vague a permis de disposer d'une large offre de dépistage lorsqu'elle a débuté. Si les deux premières vagues (virus historique) ont eu un impact sanitaire modéré (formes sévères, mortalité), cela n'a pas été le cas des troisième et quatrième (Gamma puis Delta): durant plus de 6 mois de mars à novembre 2021, environ 2 000 personnes ont été hospitalisées, dont près de 500 en soins critiques avec une mortalité hospitalière de 9,2 à 13,1% pour les vagues Gamma et Delta. Le nombre de cas détectés a été de 28 000 durant cette période mais les travaux de séroprévalence ont montré que le nombre d'immunisés était probablement 4 fois plus élevé. Comme ailleurs, la cinquième vague (Omicron) semble avoir infecté ou réinfecté beaucoup plus de personnes mais provoqué beaucoup moins de formes sévères et n'a pas saturé les services de réanimation. Il ne faut pas oublier la succession des mesures de « contrôle épidémique » impactant lourdement la société qu'ont été le confinement et les couvre-feux, avec une efficacité possible sur la dynamique épidémique sous certaines conditions, et avec certains effets délétères.

Pour un territoire jeune de 300 000 habitants doté d'une offre sanitaire limitée, le défi qu'on représenté près de 6 000 hospitalisations dont 800 en réanimation et 400 décès quasi-exclusivement hospitaliers a été majeur. L'impact de ces deux années de pandémie restera à ce jour sans égal, comparé aux nombreuses pathologies infectieuses habituelles sur le territoire, en dépit du facteur protecteur puissant de la démographie guyanaise (50% de la population a moins de 25 ans). La mortalité hospitalière observée correspond à près d'un semestre de mortalité « toutes causes » et 10 années de mortalité routière…

Pour estimer le fardeau de santé publique du Covid-19, il faut tenir compte des facteurs de vulnérabilité des populations de Guyane qui, en l'occurrence, étaient nombreux: forte prévalence du surpoids et de l'obésité morbide, du diabète (souvent méconnu ou non contrôlé) et des maladies cardiovasculaires, dont l’âge de survenue est plus précoce d'une dizaine d'années par rapport à l'Hexagone. Ainsi, les formes sévères Covid-19 étaient hospitalisées en moyenne à 50 ans, en réanimation à 60 ans, et décédaient à un âge proche de 70 ans, bien inférieur à ceux observés dans l'Hexagone. En dépit de ces fragilités, les structures de santé guyanaises, pourtant parfois éloignées d'une partie de la population, ont permis de limiter l'impact sanitaire, aidées par une politique volontariste et innovante « d'aller vers » permettant de déployer une offre de dépistage au plus près de la population, puis de vaccination, mais aussi d'oxygénothérapie au domicile. Cela a grandement contribué à éviter le désastre sanitaire malheureusement observé dans nombre d’États voisins, qui n'ont souvent pas été en mesure de massifier leur offre en soins critiques et en oxygène, lorsque la Guyane augmentait ses capacités de 500% durant plusieurs mois. Un des facteurs de résilience, outre les renforts obtenus, a aussi été l'expérience acquise sur un territoire qui connaît de longue date les pandémies récurrentes d'arboviroses.

Si le futur reste incertain, l'immunisation vaccinale reste insuffisante pour protéger les plus fragiles (un Guyanais sur deux, audelà de 50 ans, présente un schéma vaccinal complet), tandis que l'hésitation vaccinale a aussi concerné les soignants, même si la Guyane a une séroprévalence parmi les plus élevées de France (63,9% en août 2021). Selon les futurs variants qui circuleront, les plus vulnérables resteront exposés au risque de forme sévère. Ceci d'autant plus que les inégalités de santé sont de puissants déterminants de l'accès au dépistage, aux soins et à la vaccination, en particulier pour les populations les plus isolées.

# Research article: Le Covid-19 au CHU de la Martinique: saison 1 à 5

**Published:** 2022-08-29

**Authors:** André CABIE

**Affiliations:** Centre hospitalier universitaire de Martinique, 97261; Fort-de-France Cedex, France

Depuis le premier cas de Covid-19 diagnostiqué en Martinique le 5 mars 2020, les vagues épidémiques se sont succédé avec des caractéristiques épidémiologiques et des impacts sociétaux très différents. Après quelques cas venus directement d'Europe, la première vague a été caractérisée par une contamination initiale de Martiniquais à bord de bateaux de croisière embarquant et débarquant à chaque étape caribéenne des passagers venus de la Caraïbe et d'Europe. De nombreuses personnes hospitalisées ont eu à subir stigmatisation et rejet de la part de leur entourage et de certains professionnels.

La deuxième vague (09/20), plus longue, contemporaine d'une épidémie de dengue de grande ampleur, a entraîné une forte tension hospitalière.

La troisième (03/21) a immédiatement suivi la semaine de carnaval au cours de laquelle les mesures de freinage de l’épidémie ont été peu respectées. L'impact hospitalier a été plus fort que pour les deux premières vagues.

Mais c'est la quatrième vague (07/21) qui a provoqué un débordement total, alors inédit en France, du CHU et du système de santé martiniquais. À la mi-août, le CHU s’était transformé en « hôpital Covid » avec plus de 500 personnes hospitalisées simultanément et un afflux permanent de patients en détresse respiratoire. L'aide nationale a été massive: médicaments, matériels, soignants par centaines, renfort des forces armées, évacuations sanitaires vers l'Hexagone par gros-porteurs… Ce drame a pu être objectivé par un pic de mortalité sans précédent depuis un siècle. Cette quatrième vague et la cinquième (décembre 2021) ont été accompagnées de mouvements sociaux violents, et d'une défiance, parfois agressive, envers la médecine (vaccination) et les professionnels de santé.

La succession de ces vagues épidémiques a très durement et sans doute durablement éprouvé l’état de santé des Martiniquais, la société et le système de soins de la Martinique.

# Research article: Étendue de la transmission du Covid-19 dans la population guyanaise: le projet ÉPICOVID

**Published:** 2022-08-29

**Authors:** Claude FLAMAND

**Affiliations:** Institut Pasteur de la Guyane, 23 Avenue Pasteur, BP 6010, Cayenne Cedex 97306, Guyane, France

La propagation du SARS-CoV-2 s'est accompagnée, au début de la pandémie, d'une grande incertitude relative aux principales caractéristiques épidémiologiques de la transmission. Dans un contexte où la surveillance épidémiologique était majoritairement ciblée sur les patients symptomatiques, il est apparu nécessaire de mettre en place un projet visant à investiguer le spectre complet de la maladie, l’étendue réelle de la transmission en prenant en compte l'ensemble des infections, y compris les plus légères ne nécessitant pas de soins médicaux. Le projet ÉPICOVID mis en place en mars 2020 par l'Institut Pasteur de la Guyane, en collaboration avec le Centre hospitalier de Cayenne, l'Agence Santé publique France, les différents laboratoires d'analyse de biologie médicale de Guyane, les Centres de la Croix-Rouge et l'Agence régionale de santé a permis d’évaluer l’étendue de la transmission du SARSCoV-2 en Guyane, à partir d'une étude de transmission intra-ménage et d'enquêtes de séroprévalence populationnelles répétées dans le temps.

Les ménages suivis étaient inclus une fois qu'un premier cas biologiquement confirmé avait été identifié. Le suivi des ménages incluait un suivi virologique par RT-PCR à partir d’écouvillons naso-pharyngés et un suivi clinique pour l'ensemble des membres du ménage durant les 28 premiers jours suivant la date de confirmation du cas index.

Un suivi sérologique incluant 9 prélèvements sanguins répétitifs a également été réalisé afin de suivre la dynamique des anticorps des cas infectés sur une période de 12 mois. Trois enquêtes de séroprévalence réalisées du 15 au 23 juillet 2020 au moment du pic épidémique de la première vague, du 28 septembre au 9 octobre 2020 a? la fin de la première vague et du 23 août au 24 septembre dans la phase ascendante de la quatrième vague ont permis de suivre l’évolution de la transmission du virus en Guyane.

Au total, 57 logements incluant 245 individus, âgés de 1 a? 85 ans ont e?te? inclus dans l’étude intra-ménages. Les délais moyens entre la date de début de leurs symptômes et la date de confirmation du diagnostic et celle de l'inclusion dans l’étude étaient respectivement de 4,2 jours et de 7,2 jours. La transmission secondaire a pu être mise en évidence dans près des trois quarts des ménages avec des taux de transmission secondaire très importants en particulier dans les populations les plus précaires, dans la sphère du couple et de l'adulte a? l'enfant. Les études de séroprévalence populationnelles ont permis de suivre les taux de séroprévalence qui ont varié de 15% au moment du pic épidémique de la première vague épidémique à près des deux tiers de la population au début de la quatrième vague malgré un faible impact de la vaccination en Guyane.

Les résultats obtenus ont mis une évidence une importante transmission du virus en Guyane associée à un faible taux de sévérité lié à la structure de la population particulièrement jeune. Le projet a permis de fournir aux autorités de santé des données utiles pour accompagner les stratégies de prévention et de lutte, ainsi que d’évaluer l'impact des interventions mises en place au cours de la pandémie.

# Research article: Covid-19 et mortalité en Guadeloupe

**Published:** 2022-08-29

**Authors:** Bruno JARRIGE

**Affiliations:** Centre hospitalier universitaire de la Guadeloupe, Route de Chauvel, 97159 Pointe-à-Pitre, France

Les 5 vagues du Covid-19 n'ont pas épargné les DOM malgré leur éloignement et pour certains leur insularité. La mortalité hospitalière des deux régions antillaises est très forte (Guadeloupe: 246 décès hospitaliers pour 100 000 habitants; Martinique: 262); celle des deux régions de l'Océan indien plus modérée (Réunion: 91; Mayotte: 56); celle de la région Guyane intermédiaire (133), comparable à la France (176), dont le Grand Est (235) et la Nouvelle-Aquitaine (105) (Fig. 2).

**Figure 2:** Comparaison de l'incidence de la mortalité hospitalière pour 100 000 habitants à Mayotte, La Réunion, Guyane, Martinique, Guadeloupe, Nouvelle-Aquitaine, Grand Est et France hexagonale Comparison of the incidence of hospital mortality per 100,000 inhabitants in Mayotte, Reunion, Guyana, Martinique, Guadeloupe, Nouvelle-Aquitaine, Grand Est and mainland France

L’âge médian des populations antillaises (43 ans pour la Guadeloupe et 47 pour la Martinique) est un facteur de risque par rapport à l'Hexagone (41 ans). Ce critère est plutôt protecteur pour la Réunion (36 ans), la Guyane (25 ans) et Mayotte (20 ans), l’âge augmentant l'incidence des maladies chroniques à risque de formes graves.

L'incidence dans ces territoires de ces maladies chroniques est par ailleurs plus importante: le diabète, l'insuffisance rénale, la malnutrition, l'obésité…

La chronologie des vagues a eu aussi un impact. Les trois premières vagues dans les DOM ont été moins morbides qu'en Hexagone. Les mesures de freinage (confinement national, couvre-feu…) ont protégé de fait les DOM dès le début de ces vagues. Une exception a été la Guadeloupe pour sa deuxième vague qui a subi une intrusion du virus depuis l'Amérique du Nord via les îles du nord bien avant les mesures nationales de freinage déclenchées avec la déclaration plus tardive de l’état d'urgence sanitaire.

Pour la quatrième vague, les Antilles et dans une moindre mesure la Guyane, ont vécu un véritable tsunami, le variant Delta donnant des formes plus sévères mais surtout de par une absence de couverture vaccinale optimale. Les Antilles ont vécu la quatrième vague entre 10 et 30% de vaccinés 2 doses. La Guyane entre 10 et 20%; La Réunion entre 21 et 56% et l'Hexagone entre 35 et 75%. EPI-PHARE a mesuré une capacité de la vaccination à diminuer de plus de 90% la mortalité (en comparant les deux groupes vaccinés et non vaccinés et par appariement sur les autres facteurs de risque). La mortalité hospitalière aux Antilles a été entre 12 et 16 fois plus importante que dans l'Hexagone. La non-adhésion des populations à la vaccination fait l'objet de recherches sociologiques.

Pour la cinquième vague, l'incidence de la mortalité est moindre. La sévérité est moindre avec Omicron mais surtout l'immunité acquise par les populations antillo-guyanaises est très forte de-puis la fin de la quatrième vague. Mais à quel prix. La Réunion, moins touchée lors les quatre premières vagues et moins adhérente à la troisième dose booster vit une cinquième vague plus meurtrière.

# Research article: L'adaptation des médecines créoles guyanaise et haïtienne à l’épidémie de Covid-19 – réponses phytothérapeutiques et représentations

**Published:** 2022-08-29

**Authors:** Marc-Alexandre TAREAU, Guillaume ODONNE

**Affiliations:** 1 Laboratoire Écologie, évolution, interactions des systèmes amazoniens (LEEISA UAR 3456), CNRS, Université de Guyane, IFREMER, Cayenne, Guyane, France; 2 Centre d'investigation clinique (INSERM 1424), Centre hospitalier Andrée Rosemon, Cayenne, Guyane, France

L'adaptation de systèmes ethnomédicinaux à l’émergence (locale ou globale) d'une pathologie est un sujet peu exploré de l'anthropologie de la maladie. La pandémie de Covid-19 a permis de l'aborder de façon synchronique en Guyane française, en questionnant 1) les logiques d'intégration du Covid-19 dans les nosologies et représentations ethnomédicinales créoles, 2) les remèdes (y compris végétaux) utilisés à titre préventif et curatif, et 3) comment les réponses ethnomédicinales s'articulent avec la biomédecine.

Le travail a été effectué selon une double méthodologie, croisant enquêtes de terrain (entretiens semi-structurés, observation participante, discussions libres) et collecte botanique (échantillons déposés à l'Herbier de Cayenne).

Dans les nosologies créoles, le Covid-19 s'inscrit dans la macro-catégorie des « maladies à fièvre ». En effet, sur la base d'une analogie symptomatologique, cette maladie a rapidement été assimilée dans ces systèmes à d'autres pathologies provoquant de fortes fièvres. Les symptômes respiratoires sont également perçus comme qualifiants, bien que secondairement. Enfin, dans une moindre mesure, et surtout chez les plus croyants, le Covid-19 est une « maladie du diable », essentiellement du fait de la disruption globale qu'elle a causée.

En conséquence de l'inscription du Covid-19 dans cette catégorie des maladies à fièvre, les plantes amères sont largement mobilisées, tant en prévention qu'en thérapeutique. Ainsi, 2/3 des citations d'usages concernent des espèces amères, qui représentent 13 espèces parmi les 36 recensées, dont *Aristolochia trilobata*, *Artemisia annua*, *Geissospermum laeve*, *Momordica charantia*, *Neurolaena lobata, Phyllanthus amarus*, *Quassia amara* ou *Tinospora crispa*. Toujours dans une logique d'analogie symptomatologique, de nombreuses plantes utilisées ici étaient auparavant utilisées pour soigner les affections des voies respiratoires. Par exemple, *Cymbopogon citratus*, *Lantana camara* ou encore *Alpinia zerumbet* occupent une place centrale. Les sirops font partie des remèdes régulièrement cités. Si ces préparations sont classiques dans les médecines créoles, les sirops à base de plantes amères ont fait leur apparition, alliant ces dernières, réputées fébrifuges, à une forme galénique préconisée contre les affections respiratoires.

Contrairement au pluralisme habituel, un clivage fort entre biomédecine et phytothérapies a été relevé, et notamment une méfiance, sinon une défiance importante de la population vis-à-vis des vaccins. Cette opposition s'est largement matérialisée sous la forme d'un communautarisme du « eux » contre « nous », « leur vaccin » contre « nos plantes ». Cette foi dans les plantes, en prévention comme en soins, alors que les risques liés à la maladie sont minimisés, tandis que les effets secondaires des vaccins sont exagérés, est très relayée par les réseaux sociaux. Une des explications peut être cette perception de la non-nouveauté de la maladie prise en tant qu'ensemble de symptômes déjà appréhendés, ainsi que de la forte valence préventive des phytothérapies créoles.

# Research article: Utilisation de la pharmacopée locale et hésitation vaccinale en Guyane

**Published:** 2022-08-29

**Authors:** Glwadys FORSANS, Claude FLAMAND, Marc-Alexandre TAREAU

**Affiliations:** 1 Unité d’épidémiologie, Institut Pasteur de la Guyane, 23 Avenue Pasteur, BP 6010, Cayenne Cedex 97306, Guyane, France; 2 Laboratoire Écologie, évolution, interactions des systèmes amazoniens (LEEISA), 275 route de Montabo, 97300 Cayenne, Guyane, France

## Introduction

L'objectif est d'enquêter sur l'utilisation des remèdes traditionnels afin d’étudier l'ampleur de la prise de traitement, de la médication alternative, de l'utilisation des remèdes traditionnels dans la population générale et les caractéristiques sociodémographiques des consommateurs.

## Matériel et méthode

Nous avons réalisé une étude descriptive au moyen d'un questionnaire informatique de 10 minutes diffusé en ligne du 26 avril au 9 mai 2021. Les données ont été pondérées.

## Résultat

Au total, 1 295 individus ont répondu au sondage. Parmi les caractéristiques sociodémographiques, le sexe féminin, les extrêmes de l’âge (18-24 ans et plus de 75 ans) et le lieu de naissance aux abords de la Guyane sont des facteurs favorisant l'utilisation des remèdes à base de plantes médicinales. Dans la lutte contre le Covid-19, seulement 31,7% des sujets ont utilisé des remèdes traditionnels, alors qu'en général 71,4% de l’échantillon utilise régulièrement cette thérapeutique. Au total, 604 remèdes différents ont été enregistrés comme « anti-coronavirus » pour un usage majoritairement préventif. L'espèce botanique la plus fréquemment utilisée est *Quassia amara* L. (Simaroubaceae) qui représente un quart des citations.

## Discussion et conclusion

Les phytothérapies guyanaises sont vivantes: en période de crise mondiale, les populations innovent et adaptent leurs pratiques pour lutter contre une maladie nouvellement apparue. D'après la littérature, plusieurs espèces utilisées en Guyane française contre le coronavirus pourraient potentiellement avoir des effets symptomatologiques comme *Quassia Amara* (connue pour son activité antipaludique), *Neurolaena lobata*, *Alpinia zerumbet* ou encore *Tinospora crispa*. Des études complémentaires seraient intéressantes pour étudier les composantes d'intérêt qualitatif et quantitatif, les mécanismes et les effets thérapeutiques.

# Research article: La place des plantes aromatiques et médicinales (PAM) de l'ethnopharmacopée de Martinique dans la prévention et le traitement de l'infection par le SARSCoV-2

**Published:** 2022-08-29

**Authors:** Emmanuel NOSSIN

**Affiliations:** TRAMIL Martinique, Programme de recherche appliquée à l'usage populaire des plantes médicinales dans la Caraïbe

Le surgissement brutal du Covid-19, cette pandémie d'origine asiatique, ne permet pas présentement de proposer des substances actives ayant fait leurs preuves sur ce Coronavirus.

Néanmoins, il a été possible de publier un livret dès le début de la pandémie contenant des PAM validées scientifiquement pour leur effet sur l'immunodéficience comme l'arada (*Petiveria alliacea*), l'herbe à couresse (*Peperomia pellucida*) ou l'herbe à aiguille (*Bidens pilosa*).

Par ailleurs, il existe une multitude de travaux de « modélisation moléculaire » (*molecular docking study*) par le moyen de l'informatique (« *in silico* »): cette approche virtuelle calcule avec précision l'orientation préférée d'une molécule vers une autre afin de former un complexe stable.

Divers angles d'attaque majeurs ont été identifiés sur le Coronavirus dès sa modélisation en 2020. Plus d'une douzaine de PAM de notre ethnopharmacopée dont la malnommée (*Chamaesyce hirta*); le piment (*Capsicum annuum*), le bilimbi (A*verrhoa bilimbi*) ou le gingembre blanc (*Curcuma zedoaria*), se sont révélés potentiellement intéressantes pour bloquer l'enzyme (3-chymotrypsine-protéase) responsable de la multiplication du virus.

En conclusion, si aucune PAM ou substance active de notre ethnopharmacopée n'a déjà fait l'objet de validation scientifique dans le traitement curatif du SARS-CoV-2, on ne saurait pour autant écarter la survenue de découvertes dans cet immense trésor que constitue la biodiversité de la Martinique et de l'Outre-mer en général.

# Research article: Deux ans de Covid-19: évolution de la perception de la vaccination chez les soignants français au cours de la pandémie

**Published:** 2022-08-29

**Authors:** Amandine GAGNEUX-BRUNON

**Affiliations:** Centre hospitalier universitaire de Saint-Étienne, Centre hospitalier universitaire de Saint-Étienne, France

En cas de pathologie émergente, les professionnels de santé sont en première ligne et paient un lourd tribut à ces maladies transmissibles. Dès septembre 2020, l'Organisation mondiale de la santé a identifié les professionnels de santé comme un groupe prioritaire pour la vaccination. Cependant, les professionnels de santé ne sont pas immunisés contre l'hésitation vaccinale. Cette hésitation vaccinale se définit comme le fait de ne pas réaliser un vaccin, de le retarder ou de le réaliser malgré des doutes sur son efficacité et sa tolérance. Le développement rapide des vaccins contre le Covid-19 et le contexte pandémique ont probablement exacerbé les positions sur la vaccination. Pendant la première vague de Covid-19, une large majorité des soignants souhaitaient se faire vacciner alors qu'aucun vaccin n’était encore annoncé. Comme dans la population générale, les intentions de se faire vacciner ont ensuite diminué jusqu'au lancement de la campagne. Dès la première vague, d'importantes disparités ont été observées entre les différents corps de métiers: infirmiers et aides-soignants ayant des intentions de se faire vacciner bien inférieures à celles des médecins et pharmaciens. De même, les professionnels de santé libéraux étaient plus enclins à se faire vacciner. Les professionnels les plus âgés, les hommes, et les professionnels se considérant à risque d'infections ou avec des facteurs de risque de formes graves étaient plus enclins à se faire vacciner. La confiance envers les institutions et les vaccins, le conformisme social et le sens de la responsabilité collective contribuaient aux intentions de vaccination. Depuis, les professionnels de santé ont fait l'objet d'une obligation vaccinale, dont l'impact sur l'acceptabilité d'autres vaccins comme celui contre la grippe saisonnière reste à déterminer.

# Research article: Covid-19 en Guyane: un virus, un vaccin, des peuples

**Published:** 2022-08-29

**Authors:** Loïc EPELBOIN

**Affiliations:** Centre hospitalier de Cayenne, Avenue des flamboyants, Cayenne 97306, Guyane, France

La Guyane, territoire ultramarin situé en Amérique du Sud, est la région de France avec la couverture vaccinale la plus faible contre le Covid-19.

Les épidémies qui ont successivement touché ce territoire amazonien ont différé de l'Hexagone par de multiples aspects: épidémies décalées, virus et variants différents, atteinte hétérogène sur le territoire et mesures sanitaires différentes, parfois « innovantes ». Parmi ces mesures se démarquant de ce qui a été fait dans l'Hexagone, on retrouve notamment l'interruption des vols intérieurs et vers les pays voisins, en particulier le Brésil, la limitation des vols outre-Atlantique vers l'Hexagone, la fermeture des frontières fluviales et terrestres avec le Suriname et le Brésil, les confinements localisés ou généralisés, en particulier à Saint-Georgesde-l'Oyapock lors de la première vague, les couvre-feux à géométrie variable, la limitation des déplacements au sein du territoire, avec contrôles des forces de l'ordre au niveau des barrages de Regina à l'Est et d'Iracoubo à l'Ouest, l'isolement forcé des patients positifs dans des lieux éloignés dédiés (Hôtel du Fleuve à Sinnamary, collège de Saint-Georges-de-l'Oyapock, etc.). Ces mesures ont été mises en place dans un contexte de territoire multiculturel et multiethnique avec des villages parfois très enclavés et difficilement accessibles, et avec l'un des taux de précarité les plus élevés du pays.

Dans ce contexte, l'arrivée de la vaccination qui devait représenter la porte de sortie et un retour vers « la vie d'avant » a au contraire été l'objet de toutes les polémiques.

Localement les autorités de santé et les soignants ont multiplié les interventions, de façon parfois judicieuse, parfois maladroite, et les actions visant à trouver des solutions pour acheminer les vaccins vers les lieux les plus reculés de la forêt amazonienne tout en convainquant les multiples communautés qui constituent la Guyane de l'intérêt de cette vaccination.

La conviction ou au contraire l'hésitation vaccinale ont pris de multiples formes selon les lieux et les communautés, du littoral aux communes de l'intérieur, du fleuve Oyapock à la frontière brésilienne, au Maroni, à la frontière surinamienne, des « métros » aux Amérindiens, des Créoles aux Hmongs, des Bushinengués aux Brésiliens, etc. Les « fake news » se sont multipliées sur les réseaux sociaux, parfois identiques à celles retrouvées ailleurs dans le monde, et parfois très spécifiques à la population dans laquelle elles circulaient. Le pire et le meilleur ont été vus en matière de communication avec des postures délétères comme des avancées importantes dans les domaines de la communication, de la médiation en santé et de l'approche en santé communautaire.

Dans la mêlée se sont retrouvés les autorités sanitaires et administratives, les soignants, les associations, les médiateurs en santé, les épidémiologistes, les mouvements autonomistes, les mouvements antivax, les autorités religieuses, les médias locaux et nationaux, les politiques de Guyane et de l'Hexagone, des populistes de tout poil, aboutissant malgré tout à une ambiance plutôt moins délétère qu'aux Antilles, mais avec une couverture vaccinale la plus faible de France. Ces nombreux phénomènes peu connus dans l'Hexagone ont profondément bouleversé la société guyanaise et ont été présentés à l'occasion de ces journées.

# Research article: La politisation du rapport au Covid et à son vaccin en Guadeloupe

**Published:** 2022-08-29

**Authors:** Stéphanie MULOT

**Affiliations:** Université Toulouse-Jean-Jaurès, 5 Allée Antonio Machado, 31058 Toulouse, France

Cette communication vise à montrer comment le contexte politique et socioculturel de la Guadeloupe a largement déterminé la réception de la gestion politique de l’épidémie, ainsi que de la campagne de vaccination.

La montée d'un discours nationaliste, ces dernières années, y a favorisé un nationalisme sanitaire et thérapeutique, fondé sur le recours aux ressources locales (humaines et naturelles), qui repose sur le besoin d'inverser le stigmate du colonisé, construit de longue date dans le présupposé de son infériorité et de son incompétence.

Nous analysons le développement de plusieurs contre-pouvoirs qui ont dessiné une nouvelle carte de la Guadeloupe. Sur le plan politique, le paysage a été marqué par l’émergence de nouveaux partis et le renforcement de syndicats et d'activistes qui ont produit un discours extrêmement critique, parfois radical, et toujours très contestataire envers la politique très verticale du gouvernement, en convoquant une matrice explicative anticoloniale.

Sur le plan médiatique, l'interconnexion de groupes multiples sur les réseaux sociaux a facilité la large diffusion d'intox, d'infox et de fake news auxquelles une partie de la population a facilement adhéré, par un mécanisme d'identification de proximité.

Sur le plan scientifique, la création d'un Conseil scientifique caribéen et la glorification des compétences des scientifiques et médecins locaux qui s'opposaient à la vaccination, ont permis à la population de croire à une alternative qui aurait permis de se passer du vaccin. Celui-ci a d'ailleurs plus été présenté sur les réseaux sociaux dans ses potentiels effets indésirables, que dans la protection qu'il apportait aux personnes les plus vulnérables et à l'ensemble de la population.

La rhétorique de la résistance l'a ainsi emporté sur la raison sanitaire. Elle s'est inscrite dans une veine post-coloniale et a exprimé le besoin d'une partie de la population de montrer ses capacités critiques, son agentivité et son potentiel d'opposition à une politique autoritaire perçue comme une tentative contemporaine de renouvellement d'un rapport de domination et d'aliénation.

La fin de la communication présente alors la confusion et les doutes que les patients hospitalisés durant la quatrième vague ont pu exposer durant notre enquête. Alors que la plupart imaginaient initialement pouvoir se soigner avec des plantes médicinales pour un « refroidissement », ils ont constaté l’échec de la pharmacopée à traiter le « changement de corps » que provoque le Covid. Si la majorité semblait toujours aussi rétive au vaccin, une partie a pu exprimer des regrets d'avoir suivi des informations mensongères, et d'avoir hypothéqué sa vie sur des rumeurs mensongères.

# Research article: Attitudes des soignants de Guyane à l’égard du vaccin contre le Covid-19

**Published:** 2022-08-29

**Authors:** Nicolas VIGNIER, Sybille GRANIER, Kepha BRUREAU, Xavier CHARLETY, Aline DE LIGNIÈRES, Mélanie GAILLET, Loïc EPELBOIN, Maylis DOUINE

**Affiliations:** CIC INSERM 1424, Centre hospitalier de Cayenne, Avenue des flamboyants, Cayenne 97306, Guyane, France

## Introduction

En janvier 2021, les autorités françaises ont mis à disposition précocement un vaccin à ARNm. Assez vite (mi-février 2021) tous les soignants y ont eu accès et ont été encouragés à se faire vacciner. Des attitudes négatives ont été observées, dans un contexte de diffusion massive de fausses informations. Fin février 2021, seuls 679/4151 (16,4%) des professionnels étaient vaccinés.

## Méthodes

Afin de mieux appréhender l'intention vaccinale des soignants en Guyane, une enquête transversale descriptive par auto-questionnaire en ligne ou papier a été mise en place du 22/01/2021 au 26/03/2021 et diffusée à l'ensemble des professionnels de santé de Guyane par des canaux multiples.

## Résultats

En tout, 579 soignants ont répondu à l'enquête (taux de participation de 14%) dont 220 médecins (38%) et 200 infirmiers (34%), exerçant en milieu hospitalier (54%) ou en libéral (22%), de sexe féminin pour 68%, originaires de métropole (60%) ou de Guyane (20%), à risque de forme grave de Covid pour 14% et avec un antécédent de Covid-19 pour 19%. Plus de la moitié d'entre eux ont rapporté un manque de confiance sur la gestion de la pandémie.

Seuls 65% des répondants ont déclaré être vaccinés ou avoir l'intention de se faire vacciner, 11% étaient indécis et 24% ne souhaitaient pas se faire vacciner. L'intention vaccinale était plus élevée parmi les médecins (87%) que parmi les infirmiers et les autres professions de santé (50% et 46%, p < 0,001), sans différence selon le mode et le lieu d'exercice. L'opposition vaccinale était plus fréquente parmi les femmes (30%), les moins de 35 ans (37%) et parmi les professionnels de santé originaires de Guyane et des Antilles françaises (48%).

En analyse multivariée par régression logistique ordinale avec sélection descendante des variables, les facteurs associés à l'intention vaccinale étaient d’être âgé de 50-64 ans ou 65+, d’être plutôt inquiet du Covid-19 (ORa = 4,20 [1,26-1,40]), d'avoir confiance en les informations transmises par les autorités, d’être vacciné contre la grippe (ORa = 2,25 [1,02-4,96]); là où être défavorable à certaines vaccinations (ORa = 0,23 [0,13-0,41]) et être originaire de la Guyane ou des Antilles (ORa = 0,39 [0,21-0,73]) étaient négativement associés.

Les principaux arguments avancés contre la vaccination étaient le manque de transparence et d'informations adaptées, ainsi que le manque de recul sur l'efficacité et la tolérance du vaccin.

## Conclusion

La défiance vaccinale est importante chez les soignants en Guyane et revêt une dimension communautaire, à l'image des attitudes de la population guyanaise. Elle nécessite d’être mieux appréhendée et prise en compte dans les décisions de santé publique.

# Research article: Vécu de l'obligation vaccinale contre le Covid-19 par les soignants en Guyane

**Published:** 2022-08-29

**Authors:** Nicolas VIGNIER, Aline DE LIGNIÈRES, Xavier CHARLETY, Loïc EPELBOIN, Maylis DOUINE

**Affiliations:** CIC INSERM 1424, Centre hospitalier de Cayenne, Avenue des flamboyants, Cayenne 97306, Guyane, France

## Introduction

La vaccination Covid-19 a été rendue obligatoire pour l'ensemble des soignants et professions apparentées en Guyane en septembre 2021. Des interdictions d'exercer et des suspensions ont été prononcées à l'encontre des professionnels non vaccinés.

## Méthodes

Afin de mieux appréhender le vécu de l'obligation vaccinale par les soignants et les professionnels de Guyane soumis à l'obligation, une enquête transversale par auto-questionnaire en ligne a été menée à compter en mars 2022 et diffusée à l'ensemble des professionnels de santé de Guyane par des canaux multiples.

## Résultats

Deux jours après la diffusion du questionnaire, 102 professionnels avaient déjà répondu à l'enquête: 75% médecins, 12% paramédicaux et 8% administratifs. Leur mode d'exercice était hospitalier (66%) ou libéral (21%), sur l’Île de Cayenne (61%), Savanes (15%) ou l'Ouest guyanais. Ils étaient des femmes à 53%, originaires de métropole (74%), de Guyane ou des Antilles (9%), ou d'autres pays (16%), à risque de forme grave de Covid-19 pour 13%, avec un antécédent de Covid-19 pour 45% et vaccinés contre le Covid-19 à 98%.

Huit pour cent des répondants n'auraient pas réalisé la vaccination si elle n'avait pas été rendue obligatoire et seuls 69% étaient favorables à l'obligation pour tous les soignants (13% pour certains professionnels seulement et 10% pour aucun). Seuls 42% ont trouvé l'information accompagnant l'obligation vaccinale suffisante et 67% ont eu le sentiment qu'elle était imposée sans concertation. Par ailleurs, 39% jugent les mesures prises contre les professionnels non vaccinés trop sévères et 31% étaient contre l'intégration du rappel dans l'obligation vaccinale.

Les verbatims relevés en faveur de l'obligation vaccinale étaient relatifs à l’évidence scientifique, la nécessité de protéger le patient et le besoin de sortir de la crise. Ceux en opposition étaient la remise en question de la liberté de choix, une décision perçue comme politique, une mise en contradiction de l'obligation avec les échecs de communication et de gestion de crise de la part des autorités, un risque infectieux perçu comme exagéré, l'existence d'intérêts financiers, le manque de moyens alloués au milieu de la santé, la mise en danger des professionnels avec un vaccin pour lequel le recul serait insuffisant et l'absence d'efficacité de la vaccination sur le risque de contamination et de transmission du SARS-CoV-2.

## Conclusion

Ces résultats préliminaires soulignent à la fois une adhésion d'une partie des professionnels à l'obligation vaccinale contre le Covid-19, mais en même temps un regard critique sur sa mise en place, traduisant ainsi un éloignement ressenti des décideurs et des professionnels de terrain.

# Research article: Caractérisation et suivi des hésitations vaccinales dans la population guyanaise

**Published:** 2022-08-29

**Authors:** Claude FLAMAND

**Affiliations:** Institut Pasteur de la Guyane, 23 Avenue Pasteur, BP 6010, Cayenne Cedex 97306, Guyane, France

Pas de résumé disponible.

# Research article: Accéder à l'eau en temps de crise sanitaire: le service public d'eau potable à l’épreuve du Covid-19 en Guyane

**Published:** 2022-08-29

**Authors:** Priscilla THEBAUX

**Affiliations:** CNRS, Laboratoire Écologie, évolution, interactions des systèmes amazoniens (LEEISA), 275 route de Montabo, 97300 Cayenne, Guyane, France

## Introduction

« Se laver les mains » est l'un des gestes barrières recommandés pour lutter contre le Covid-19, mais comment faire quand on n'a pas accès à l'eau? Pour permettre aux habitants des quartiers informels sans accès à l'eau potable du littoral de satisfaire ce besoin, les autorités publiques en Guyane ont décidé, dès mars 2020, la mise en place d'un dispositif gratuit d'accès à l'eau potable. Dans un contexte où le manque d'accès est considéré comme chronique (plus de 15% de la population n'avait pas accès à un point d'eau potable à l'intérieur du logement en 2018 selon l'INSEE), nous nous demandons à quels enjeux multiples ce dispositif vient répondre et quel est son impact dans la construction du service public en Guyane, tant du point de vue des acteurs de l'eau que des usagers.

## Matériel et méthode

Notre réflexion s'appuie sur une enquête exploratoire de terrain réalisée en 2020 auprès des habitants de quatre quartiers ainsi que des acteurs en charge du service.

## Résultats

Nous avons montré comment, dans ce contexte d'urgence, les acteurs « classiques » du service public ont tenu un rôle secondaire, laissant la place à de nouveaux acteurs. De fait, la mise en place de ces points d'eau revêt une forme singulière sur le territoire, dépendamment des choix techniques mais aussi politiques des acteurs mobilisés. Dans les usages, le dispositif vient aussi répondre à des enjeux variés, qu'ils soient d'ordre pratique ou symbolique, audelà du simple besoin de se laver les mains.

## Discussion

Si le point de départ de l'installation du dispositif est officiellement de lutter contre la propagation du virus, il doit désormais s'insérer dans des considérations propres au service public d'eau potable avec ses objectifs et ses contraintes.

# Research article: Saint-Georges-de-l'Oyapock à l’épreuve de la pandémie de Covid-19: habiter la frontière France-Brésil au temps des fermetures et des gestions de crise (2021)

**Published:** 2022-08-29

**Authors:** Margot SCHNEIDER, Damien DAVY, Denis CHARTIER

**Affiliations:** Université de Paris, Laboratoire Écologie, évolution, interactions des systèmes amazoniens (LEEISA), 275 route de Montabo, 97300 Cayenne, Guyane, France; LabEx DRIIHM & Observatoire hommes-milieux CNRS

## Introduction

L’étude vise à appréhender les impacts de la crise sanitaire sur les pratiques et la vie des habitants de Saint-Georges-del'Oyapock, à la frontière franco-brésilienne. La nécessité d'endiguer l’épidémie a placé la région, pourtant marginalisée, au coeur des politiques sécuritaires, soulevant des questions quant aux conséquences sociales, économiques et sur la mobilité.

## Méthode

Une méthodologie qualitative et quantitative a été choisie, mêlant entretiens compréhensifs (31), cartes mentales (15) et comptages de pirogues.

## Résultats

À Saint-Georges, la crise sanitaire a été doublée d'une gestion sécuritaire de la frontière-fleuve. Les positions politiques nationales autour de la question de la frontière et de la propagation du virus sont inhérentes à la compréhension des choix qui ont été appliqués localement à Saint-Georges. Cet espace, isolé spatialement et socio-économiquement, a subi une augmentation de la précarité de ses habitants du fait de la réduction de leur mobilité. L'inégalité déjà présente entre les habitants s'est fait davantage ressentir du fait de la pandémie, puisque peu d'entre eux pouvaient se permettre de s'approvisionner dans les supérettes du bourg où les prix sont bien plus élevés qu'au Brésil.

## Discussion et conclusion

Cette crise sanitaire a révélé plusieurs phénomènes sur l'Oyapock. La gestion sécuritaire, avec le déploiement de l'appareil coercitif de l’État à la frontière et aux abords du dispensaire, a soulevé des questions d'ordre médical, opposant notamment certains soignants aux institutions politiques. Si la frontière s'est révélée incontrôlable, les Oyapockois qui ont continué à traverser le fleuve pour s'approvisionner au Brésil n'avaient pas le choix financier de faire autrement, s'exposant ainsi à d'autres préjudices (amendes, saisies…). Les habitants ont su faire preuve d'adaptation (sous la contrainte), en se servant de leurs réseaux sociaux et familiaux, mais ont pour autant développé un sentiment général d'isolement et d'abandon de la part des institutions.

# Research article: Déferlantes, ressac et moutons: quels indices psycho-sociétaux de la marée Covid-19 en Guyane?

**Published:** 2022-08-29

**Authors:** Frédérique GROENE

**Affiliations:** Laboratoire Migrations, interculturalité et éducation en Amazonie (MINEA), EA 7485, Université de Guyane, Cayenne 97300, Guyane, France

Proposer un pas de côté, une mise en perspective basés sur des éléments d'analyse issus des sciences compréhensives, tels sont les apports de l'anthropologie médicale qui alimentent l'idée que ce n'est pas un virus seul qui crée une pandémie, mais les chaînes d'actions, de réactions individuelles et sociétales qu'il génère. La littérature scientifique met en exergue des invariants et des récurrences au niveau social et psychologique dans les épisodes de crises épidémiques.

L'attention a été portée spécifiquement sur les moyens mobilisés autour de la veille « santé mentale » en Guyane depuis le début de la crise: délestage du 15 vers une écoute spécifique par des infirmiers en psychiatrie, mobilisation des psychologues du Centre hospitalier Andrée Rosemon auprès des équipes soignantes, numéro vert d’écoute « Covid » AGP1-CTG, ainsi que les dispositifs médico-sociaux « allant vers » les populations les plus en difficulté.

1) Association guyanaise des psychologues / Collectivité territoriale de Guyane (AGP/CTG);
2) Territoires, populations et Covid-19 en Guyane: le cas de deux foyers communautaires (le village Cécilia et le kampoe Tonka);
3) Communautés amérindiennes et noirmarrons de Guyane vivant dans les anciens clusters.

Enfin, a été abordée la recherche TEPCov2 en cours, et présentés les résultats préliminaires de l'axe 6. Enquête par autoquestionnaire en ligne, organisé en 4 grands axes: la perception de la crise Covid-19, les facteurs individuels, le vivre-ensemble et la projection vers le futur. Les premiers résultats esquissent une forte mise en tension du vivre-ensemble, un affaiblissement des figures d'autorité historiques, ainsi que l'existence d'un état d'anxiété latente dans l’échantillon.

La poursuite de l'enquête au sein des communautés Lokono et Djuka, le croisement des données et leur analyse permettront de qualifier l'impact spécifique de la crise Covid-19 en Guyane.
